# Analysis of atmospheric temperature data by 4D spatial–temporal statistical model

**DOI:** 10.1038/s41598-021-98125-2

**Published:** 2021-09-21

**Authors:** Ke Xu, Yaqiong Wang

**Affiliations:** 1 School of Statistics, University of International Business and Economics, Beijing, China; 2 Guanghua School of Management, Peking University, Beijing, China

**Keywords:** Applied mathematics, Atmospheric dynamics

## Abstract

The meteorological data such as temperature of the upper atmosphere is ssential for accurate weather forecasting. The Universal Rawinsonde Observation Program (RAOB) establishes an extensive radiosonde network worldwide to observe atmospheric meteorological data from the surface to the low stratosphere. The RAOB data data has very high accuracy but can offer a very limited spatial coverage. Meanwhile, ERA-Interim reanalysis data is widely available but with low-quality. We propose a 4D spatiotemporal statistical model which can make effective inferences from ERA-Interim reanalysis data to RAOB data. Finally, we can obtain a huge amount of RAOB data with high-quality and can offer a very wide spatial coverage. In empirical research, we collected data from 200 launch sites around the world in January 2015. The 4D spatiotemporal statistical model successfully analyzed the observation gaps at different pressure levels.

## Introduction

Being able to accurately understand different temperatures at different latitudes, longitudes and altitudes is very important for research and practical applications^1–4^. These temperatures have very important applications in agriculture, ecology, biology, medicine, construction engineering, etc.^5–9^. The observation of Earth’s temperatures from space is generally recognized to be the key to future climate understanding. To ensure the temperature data from satellite products meet the mission specific requirements for climate and weather applications, rigorous validation and uncertainty assessment relies on in-situ observations, especially on radiosonde^10–12^.

One source of temperature data is from a project on Gap Analysis for Integrated Atmospheric ECV Climate Monitoring (http://www.gaia-clim.eu). It introduced the concept of network gap, which is a region of the atmosphere where the network forecasting capability is poor. This gap is relevant for both satellite validation and climate change understanding. One of this kind of data is ERA-Interim reanalysis data provided by European Centre for Medium-range Weather Forecasts (ECMWF).

Relative to ERA-Interim reanalysis data, reference measurements such as those provided by GCOS (Global Climate Observing System) Reference Upper Air Network (http://www.gruan.org) are the ideal counterparts for satellite validation. In practice, data of the Universal RAwinsonde OBservation program (http://www.raob.com) are often used as a de-facto standard for this purpose. We abbreviate this kind of temperature data as RAOB data. RAOB data is very accurate temperature data. However, RAOB data offer a very limited spatial coverage. Moreover, RAOB data is usually very expensive and difficult to obtain.

In conclusion, ERA-Interim reanalysis data is widely available with low-quality; RAOB data with high-quality but can offer a very limited spatial coverage. Therefore, it is very important to establish a connection between these two kinds of temperature data. It can help scientists make effective inferences from ERA-Interim reanalysis data to RAOB data. This gives a new way to obtain RAOB data which is expensive and difficult to collect. To this end, we propose a 4D (3D space + 1D time) statistical model, it aims to estimate the observation gap between ERA-Interim reanalysis data and RAOB data. The 4D model merges two building blocks: a spatiotemporal model on the sphere, and the functional data approach. In fact on the one side, spatiotemporal statistical modelling of global atmospheric data must be able to consider both the spherical domain and the anisotropy of the atmospheric dynamics. Moreover, radiosonde data arise as atmospheric profiles and a "natural" statistical framework is based on functional data approach^13,14^. The second building block is based on the functional representation of atmospheric profiles. In this regard, the ideas of Giraldo^15^ developed in the last ten years to spatial functional data and functional kriging. A fruitful approach is based on the representation of random functional objects as linear combinations of the basis functions with Gaussian random coefficients^16^.

Thanks to this approach, in this paper, we consider the RAOB temperature profiles indexed in space according to the launching site and indexed in time according to the day as mixed effect models with a vertical dimension given by B-spline basis functions. The spline coefficients, indexed in space and time, are modelled as spatiotemporal models on the sphere. In fact, we use an extension of the observation-minus-background approach, which is common in atmospheric sciences^17,18^ and filter out large scale components.

The rest of the paper is organized as follows. “Datasets” section describes the RAOB dataset, and the ERA-interim reanalysis data, which is used as the background. In “4D spatiotemporal statistical model” section, we present the 4D spatio-temporal statistical model. “Model results” section reports the model results, and “Conclusion” section concludes.

## Datasets

In this section, we introduce the data used for the estimation and validation of the model: the RAOB radiosonde temperature vertical profiles which are used as the model response, and the European Centre for Medium-range Weather Forecasts (ECMWF) ERA-interim reanalysis data, which are used as the background.

### The universal rawinsonde observation program

In this paper, we use the RAOB dataset for January, 2015. This dataset is obtained from the database implemented by the Earth System Research Laboratory of the National Oceanic and Atmospheric Administration (NOAA/ESRL, https://ruc.noaa.gov/raobs/).

**Figure 1:**
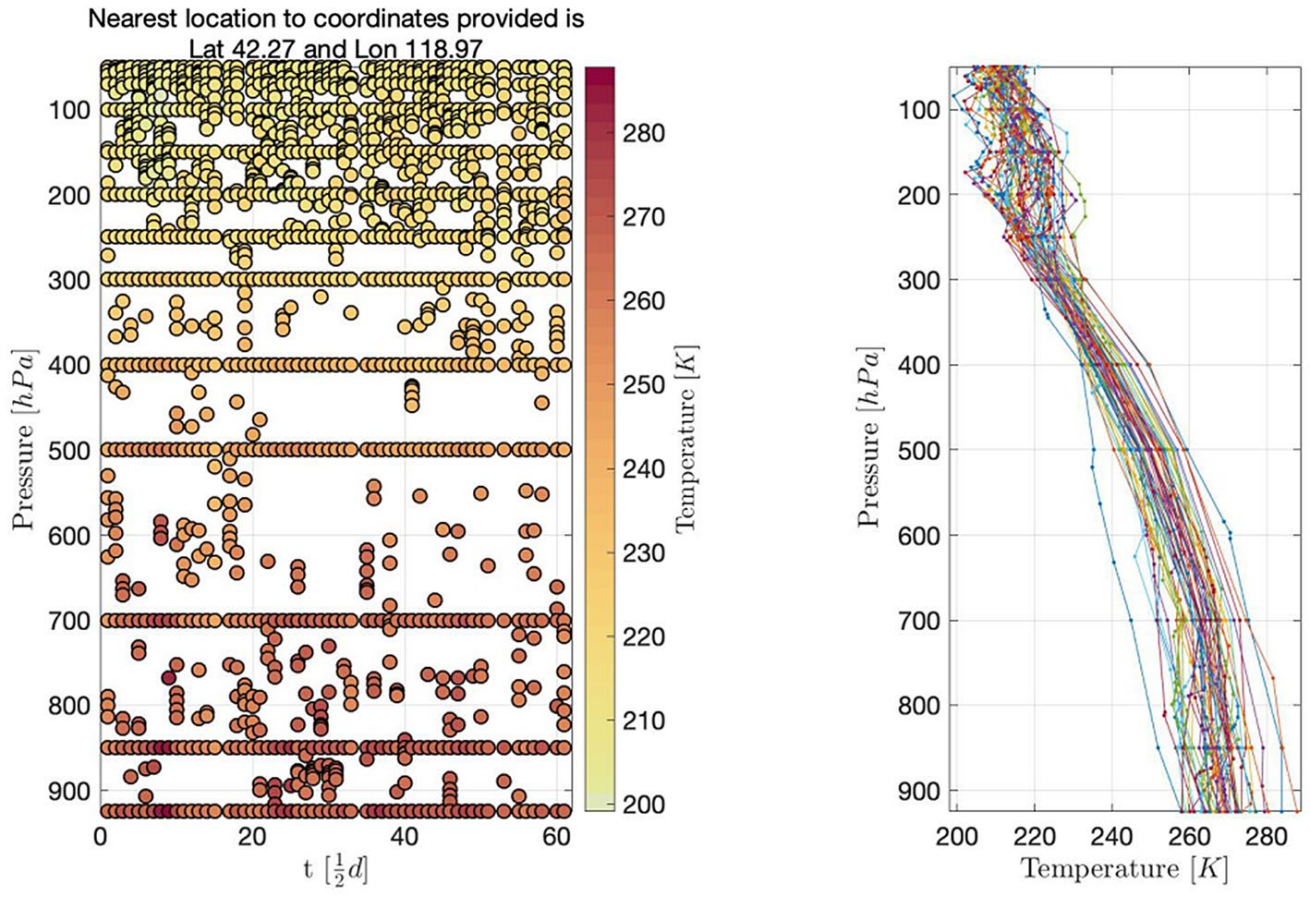
In the left panel, the vertical axis on the left represents pressure, and the vertical axis on the right represents temperature. The horizontal axis represents time points, and data are collected every half day. In the right panel, the vertical axis represents pressure, and the horizontal axis represents temperature. Data collected at different times are represented by lines of different colors. All data are collected at the latitude 42.27 and longitude 118.97 in January 2015.

We consider twice a day observations at 00:00 UTC and 12:00 UTC from the $\mathrm{n}=200$ worldwide launch sites. Temperature data collected at the latitude 42.27 and longitude 118.97 in January 2015 is depicted in Fig. 1, where the y-axis presents the barometric pressure levels.

A radiosonde is made by a set of instruments carried into the atmosphere by a weather balloon, measuring various atmospheric parameters and transmitting them by radio to a ground receiver up to altitudes of approximately 30–35 km, depending on the balloon burst. The observations are processed and encoded for transmission and efficient storage. While the radiosonde transmits an essentially continuous stream of temperature readings back to the station (each 5–10 m of altitude, measured each 1–2 s), only a subset of this information is encoded and transmitted.

### ERA-interim description

ERA-Interim is a global atmospheric reanalysis data provided by ECMWF. It is based on assimilating global observations into a computational model which gives the best state estimate^19^. We focus on 00:00 UTC and 12:00 UTC reanalysis which are described as instantaneous, though they represent 30 min averages and have a horizontal resolution of ${0.75}^{\mathrm{o}}$, which is about $80 \; \mathrm{ km}$ depending on latitude. The product comprises 60 vertical levels from the surface up to $0.1 \; \mathrm{ hPa},$ and each level is populated by 241 $\times$ 480 pixels.

## 4D spatiotemporal statistical model

In this section, we introduce a 4D spatiotemporal statistical model, which fits the functional data on the sphere. Let ${\mathrm{y}}_{\mathrm{TEMP}}\left(\mathrm{s},\mathrm{h},\mathrm{t}\right)$ be the atmospheric profile at site with coordinates $\mathrm{s}=\left(\mathrm{lat},\mathrm{lon}\right)\in {\mathbb{S}}^{2}$ and time $\mathrm{t}$, observed at altitude or barometric pressure $\mathrm{h}$, where ${\mathbb{S}}^{2}$ is the sphere representing the Earth. The model is defined by the following measurement and dynamics equations:

1 $$\begin{array}{c}{\mathrm{y}}_{\mathrm{TEMP}}\left(\mathrm{s},\mathrm{h},\mathrm{t}\right)={\mathrm{x}}_{\mathrm{ERA}}\left(\mathrm{s},\mathrm{h},\mathrm{t}\right){\upbeta }_{\mathrm{ERA}}\left(\mathrm{h}\right)+\upphi {\left(\mathrm{h}\right)}^{{{\prime}}} \text{z}\left(\mathrm{s},\mathrm{t}\right)+\upepsilon \left(\mathrm{s},\mathrm{h},\mathrm{t}\right),\end{array}$$

2 $$\begin{array}{c} \text{z}\left(\mathrm{s},\mathrm{t}\right)=\text{G}\text{z}\left(\mathrm{s},\mathrm{t}-1\right)+\upeta \left(\mathrm{s},\mathrm{t}\right).\end{array}$$

In this model, ${\mathrm{y}}_{\mathrm{TEMP}}\left(\mathrm{s},\mathrm{h},\mathrm{t}\right)$ represents RAOB data and ${\mathrm{x}}_{\mathrm{ERA}}\left(\mathrm{s},\mathrm{h},\mathrm{t}\right)$ represents ERA-Interim reanalysis data. Among (1), ${\varvec{s}}$ represents different position of launching site on the two-dimensional plane of longitude and latitude, $h$ represents the third dimension of altitude or pressure, and $t$ represents the fourth dimension of time. In addition, $\upphi {\left(\mathrm{h}\right)}^{{{\prime}}}\mathrm{z}\left(\mathrm{s},\mathrm{t}\right)$ means the difference between RAOB data and ERA-Interim reanalysis data with altitude $h$ in position ${\varvec{s}}$ at time $t$. To this end, this is a 4D model, which is relatively novel. This model is very different from conventional 3D models in traditional literature. In the case study, we define $\mathrm{h}\in \mathcal{H}=\left[50, 925\right] \; \mathrm{hPa}$, $\mathrm{t}=1,\dots ,62$. In the measurement equation, $\upepsilon \left(\mathrm{s},\mathrm{h},\mathrm{t}\right)$ is a Gaussian measurement error. The variance of $\upepsilon \left(\mathrm{s},\mathrm{h},\mathrm{t}\right)$ is represented as ${\upsigma }_{\upepsilon }^{2}\left(\mathrm{h}\right).$ To this end, ${\upsigma }_{\upepsilon }^{2}\left(\mathrm{h}\right)$ needs to satisfy the following condition:

3 $$\begin{array}{c}\mathrm{log}\left({\upsigma }_{\upepsilon }^{2}\left(\mathrm{h}\right)\right)= \upphi {\left(\mathrm{h}\right)}^{{{\prime}}}{\mathrm{c}}_{\upepsilon },\end{array}$$

where $\upphi (\mathrm{h})$ is a set of $\mathrm{p}\times 1$ basis functions, and ${\upbeta }_{\mathrm{ERA}}\left(\mathrm{h}\right)$ is modelled as:

4 $$\begin{array}{c}{\upbeta }_{\mathrm{ERA}}\left(\mathrm{h}\right)=\upphi {\left(\mathrm{h}\right)}^{{{\prime}}}{\mathrm{c}}_{\upbeta },\end{array}$$

where ${\mathrm{c}}_{\upbeta }$ is a $\mathrm{p}\times 1$ vector needed to be estimated. In Eq. (2), $\mathrm{z}\left(\mathrm{s},\mathrm{t}\right)$ is a $\mathrm{p}$-variate latent random variable with Markovian dynamics ruled by the persistency matrix $\mathrm{G}$. Therefore, G matrix is the correlation coefficient matrix of latent random variable z(s,t) in time. In the dynamics equation, $\upeta$(s; t) is a $\mathrm{p}$ dimensional Gaussian innovation random field which is independent in time, with spatial covariance function:

5 $$\begin{array}{c}\Gamma \left(\mathrm{s},{\mathrm{s}}{{^{\prime}}};\uptheta \right)=\text{diag}\left({\mathrm{v}}_{1}\uprho \left(\mathrm{s},{\mathrm{s}}^{{{\prime}}};{\uptheta }_{1}\right),\dots ,{\mathrm{v}}_{\mathrm{p}}\uprho \left(\mathrm{s},{\mathrm{s}}^{{{\prime}}};{\uptheta }_{\mathrm{p}}\right)\right).\end{array}$$

In fuction (5), $\mathrm{v}=({\mathrm{v}}_{1},\dots ,{\mathrm{v}}_{\mathrm{p}}){^{\prime}}$ is the variance vector, and $\uprho \left(\mathrm{s},{\mathrm{s}}^{{{\prime}}};{\uptheta }_{\mathrm{j}}\right)$ is a valid spatial correlation function^20^ on the sphere with range parameter ${\uptheta }_{\mathrm{j}}$ from $\uptheta =({\uptheta }_{1},\dots ,{\uptheta }_{\mathrm{p}}){^{\prime}}$. In addition, “diag()” means the elements on the diagonal of the matrix.

The $\mathrm{O}-\mathrm{B}$ approach is generalized here by using the ERA-interim background as the unique covariate of model, where O refers to observations and B refers to background^21^. In fact, the $\mathrm{O}-\mathrm{B}$ approach is generalized to $\mathrm{O}=\mathrm{\beta B}+\mathrm{e}$, where $\mathrm{e}={\Phi }^{{{\prime}}}\mathrm{z}+\upepsilon$ is capable to give a more detailed description of the bias structure. To model the effect of the East–West atmospheric dynamics on the $\mathrm{O}-\mathrm{B}$ error, the following simple anisotropic correlation structure is used:

6 $$\begin{array}{c}\uprho \left(\mathrm{s},{\mathrm{s}}^{{{\prime}}};\uptheta \right)=\mathrm{exp}\left(-\frac{\mathrm{d}\left(\mathrm{s},{\mathrm{s}}^{{{\prime}}}\right)}{{\uptheta }^{\left(1\right)}}\right)\mathrm{exp}\left(-\frac{\left|{\mathrm{l}}_{1}-{\mathrm{l}}_{1}{^{\prime}}\right|}{{\uptheta }^{\left(2\right)}}\right).\end{array}$$

**Figure 2:**
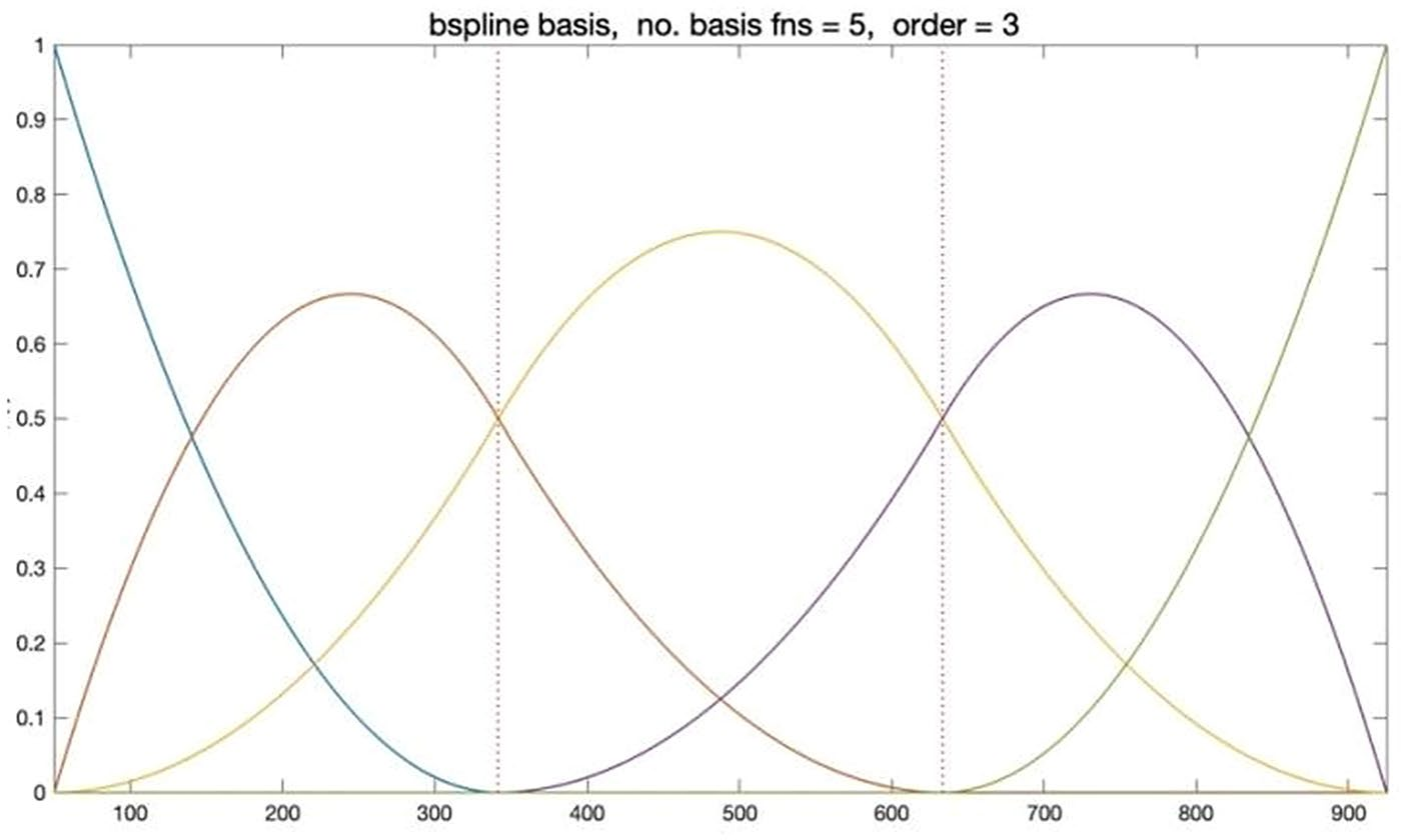
Example of a B-spline basis with 5 functions represented by 5 different colors.

In this paper, we use the B-spline system (B-spline) as $\upphi (\mathrm{h})$ in the above model equation. Knots are the values of where the pieces of polynomial meet, which are denoted and sorted into nondecreasing order. Figure 2 shows a B-spline curve with an order of 3 and knots of 3, with the vertical dashed line as the internal breakpoint that defines the spline curve. Note that the sum of the B-spline basis function values at any point $\mathrm{h}$ is equal to 1.

## Model results

We first estimated the model by Expectation–Maximization algorithm. Second, we analyzed the model estimation results and verified the fitting goodness of our model based on cross-validation ${\mathrm{R}}^{2}.$ Finally, based on the results of spatial prediction, we analyzed the observation gaps by $\mathrm{O}-\mathrm{B}$ approach.

### Model estimation results

We choose Bspline to model Eqs. (1–2). As the accuracy of the data at high altitude decreases, we set more internal breakpoints at high altitudes where the barometric pressure is below $300 \; \mathrm{ hPa}$. Based on the empirical analysis, we set the parameter knots for estimating ${\upbeta }_{\mathrm{ERA}}\left(\mathrm{h}\right)$ and ${\upsigma }_{\upepsilon }^{2}\left(\mathrm{h}\right)$ as

$$\mathrm{knots}=\left[50, 150, 210, 290, 380, 510, 700, 925\right].$$

The estimated coefficient $\mathrm{z}\left(\mathrm{s},\mathrm{t}\right)$ of the latent term $\upphi {\left(\mathrm{h}\right)}{{^{\prime}}}\mathrm{z}\left(\mathrm{s},\mathrm{t}\right)$ in Eq. (1) is p-variate latent Gaussian random variable. Since the computation burden would be pretty high if too many knots are set, here the knots is as

$$\mathrm{knots}=\left[50, 340, 925\right].$$

We implement a cubic spline here. Therefore, the number of basis functions for ${\upbeta }_{\mathrm{ERA}}\left(\mathrm{h}\right)$, ${\upsigma }_{\upepsilon }^{2}\left(\mathrm{h}\right)$ and $\upphi {\left(\mathrm{h}\right)}^{{{\prime}}}\mathrm{z}\left(\mathrm{s},\mathrm{t}\right)$ is 9, 9, 4 respectively. Tables 1 and 2 show the estimate results for ${\mathrm{c}}_{\upbeta }$ and ${\mathrm{c}}_{\upepsilon }$.

**Table 1:** Result of ${\mathrm{c}}_{\upbeta }$ estimates.

${\mathrm{c}}_{\upbeta }$	Estimates	Std. error	t-statistic
Basis_1_@ERA	0.999	0.000	6709.991
Basis_2_@ERA	1.000	0.000	6045.506
Basis_3_@ERA	0.999	0.000	8770.185
Basis_4_@ERA	0.998	0.000	9224.893
Basis_5_@ERA	1.000	0.000	10,071.891
Basis_6_@ERA	0.999	0.000	10,766.577
Basis_7_@ERA	0.999	0.000	9800.519
Basis_8_@ERA	0.999	0.000	8499.463
Basis_9_@ERA	1.000	0.000	8200.708

**Table 2:** Result of ${\mathrm{c}}_{\upepsilon }$ estimates.

${\mathrm{c}}_{\upepsilon }$	Estimates	Std. error	t-statistic
Basis_1	1.444	0.015	93.849
Basis_2	1.137	0.026	44.112
Basis_3	0.519	0.021	24.539
Basis_4	0.024	0.025	0.972
Basis_5	0.499	0.029	17.490
Basis_6	0.519	0.024	21.602
Basis_7	0.004	0.024	0.165
Basis_8	0.790	0.028	28.027
Basis_9	0.400	0.022	18.005

**Figure 3:**
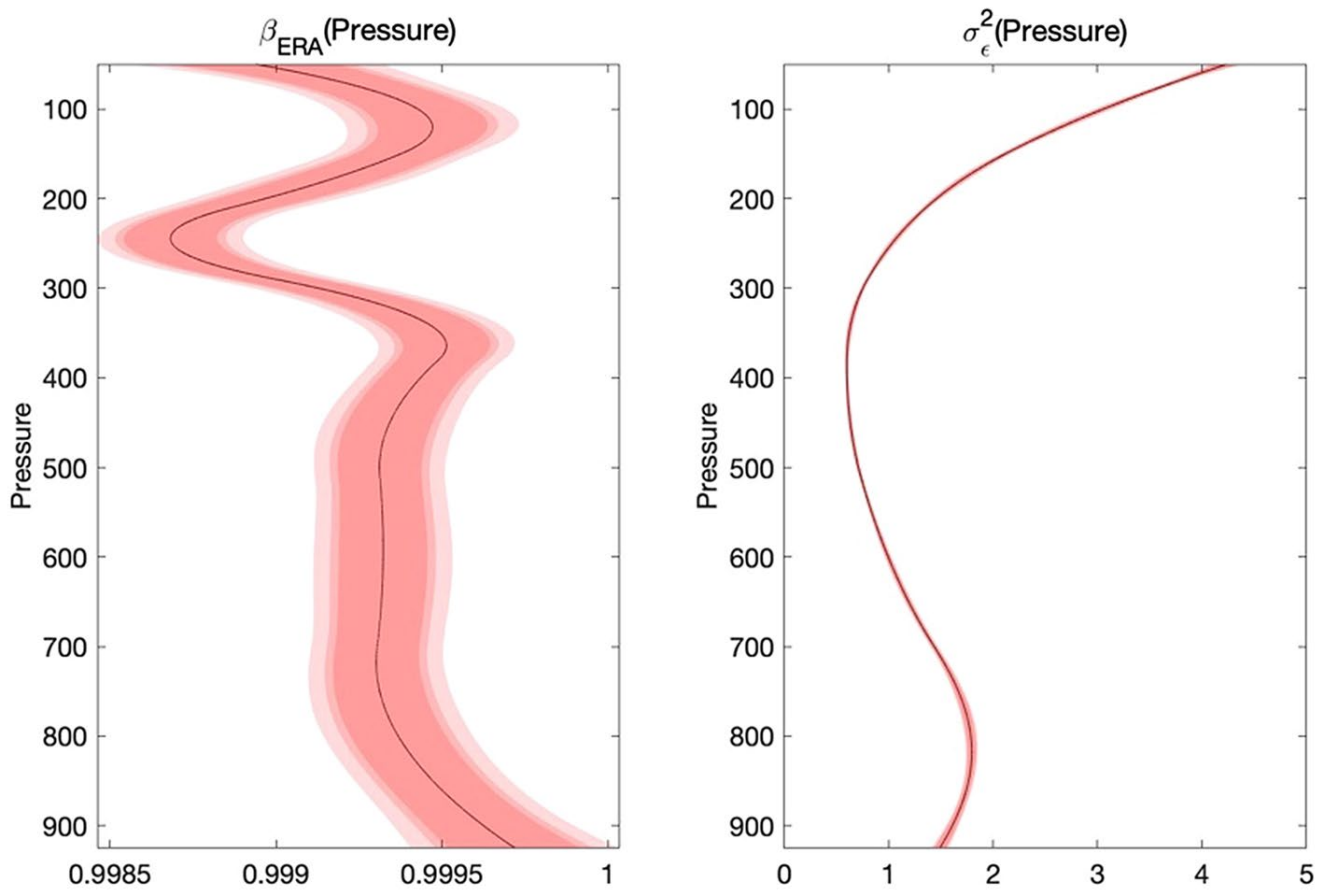
${\upbeta }_{\mathrm{ERA}}\left(\mathrm{h}\right)$ estimate and its 90%, 95%, 99% confidence bands (left), ${\upsigma }_{\upepsilon }^{2}\left(\mathrm{h}\right)$ estimate and its 90%, 95%, and 99% confidence bands (right). Among them, the outermost light red border on both sides represent 90% confidence bands, red border in the middle of both sides represent 95% confidence bands, and the innermost crimson border on both sides represent 99% confidence bands.

We plot the ${\upbeta }_{\mathrm{ERA}}\left(\mathrm{h}\right)$ and ${\upsigma }_{\upepsilon }^{2}\left(\mathrm{h}\right)$ estimates in Fig. 3. We can find that in the low-altitude layer with barometric pressure higher than $400 \;\mathrm{ hPa}$, the estimated value of ${\upbeta }_{\mathrm{ERA}}\left(\mathrm{h}\right)$ is close to 1, and the variance ${\upsigma }_{\upepsilon }^{2}\left(\mathrm{h}\right)$ of the residuals is pretty low. In the atmosphere where the barometric pressure is lower than $400 \; \mathrm{ hPa}$, the variance ${\upsigma }_{\upepsilon }^{2}\left(\mathrm{h}\right)$ of the residuals increases, and ${\upbeta }_{\mathrm{ERA}}\left(\mathrm{h}\right)$ deviates from the value 1. Thus, our model captures the bias from high-level RAOB temperature data, benefiting the accuracy of estimation of parameters.

### Model fitting goodness

**Figure 4:**
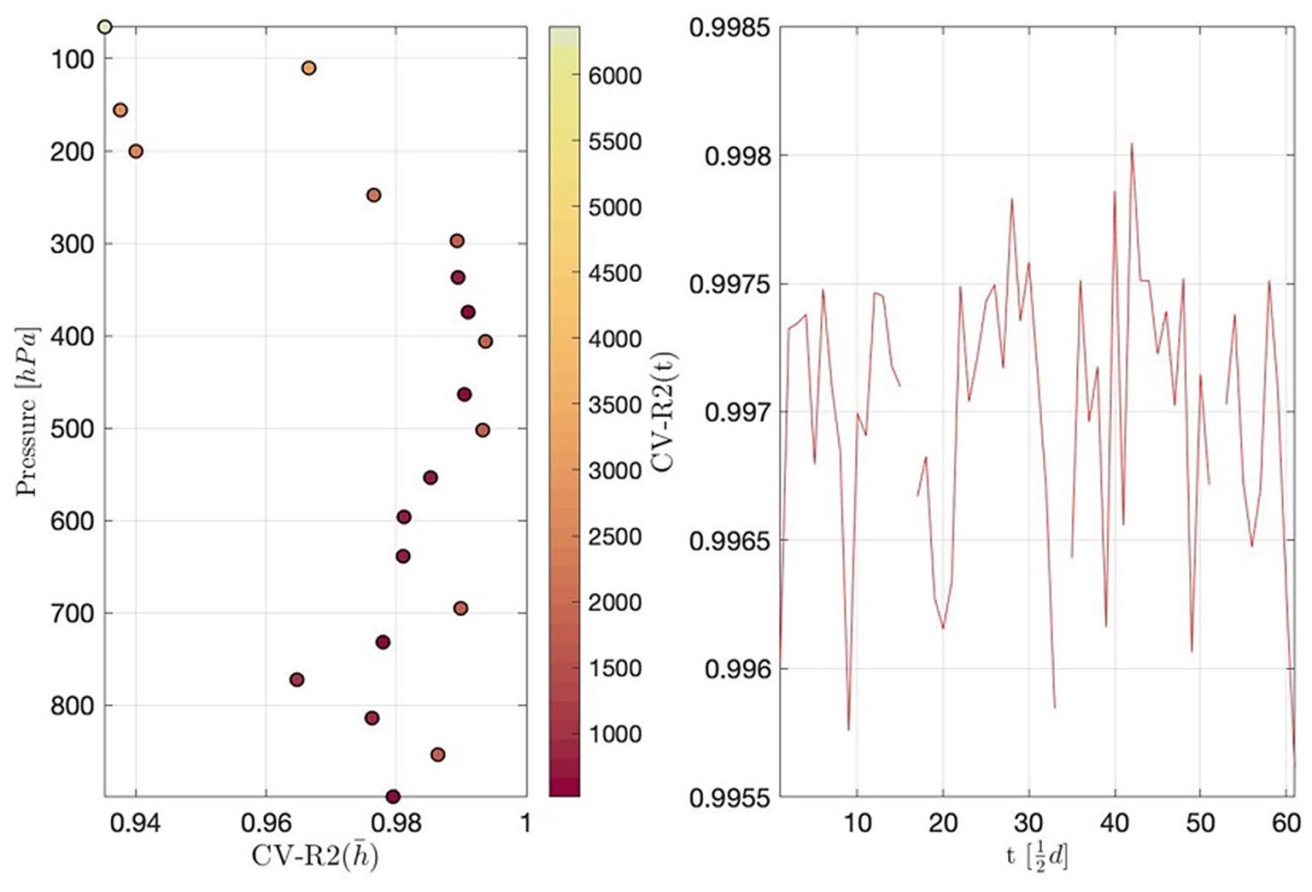
In the left panel, the horizontal axis represents cross-validation ${\mathrm{R}}^{2}$ with respect to h and the vertical axis represents pressure. In the right panel, the horizontal axis represents different time and the vertical axis represents cross-validation ${\mathrm{R}}^{2}$ with respect to t.

**Figure 5:**
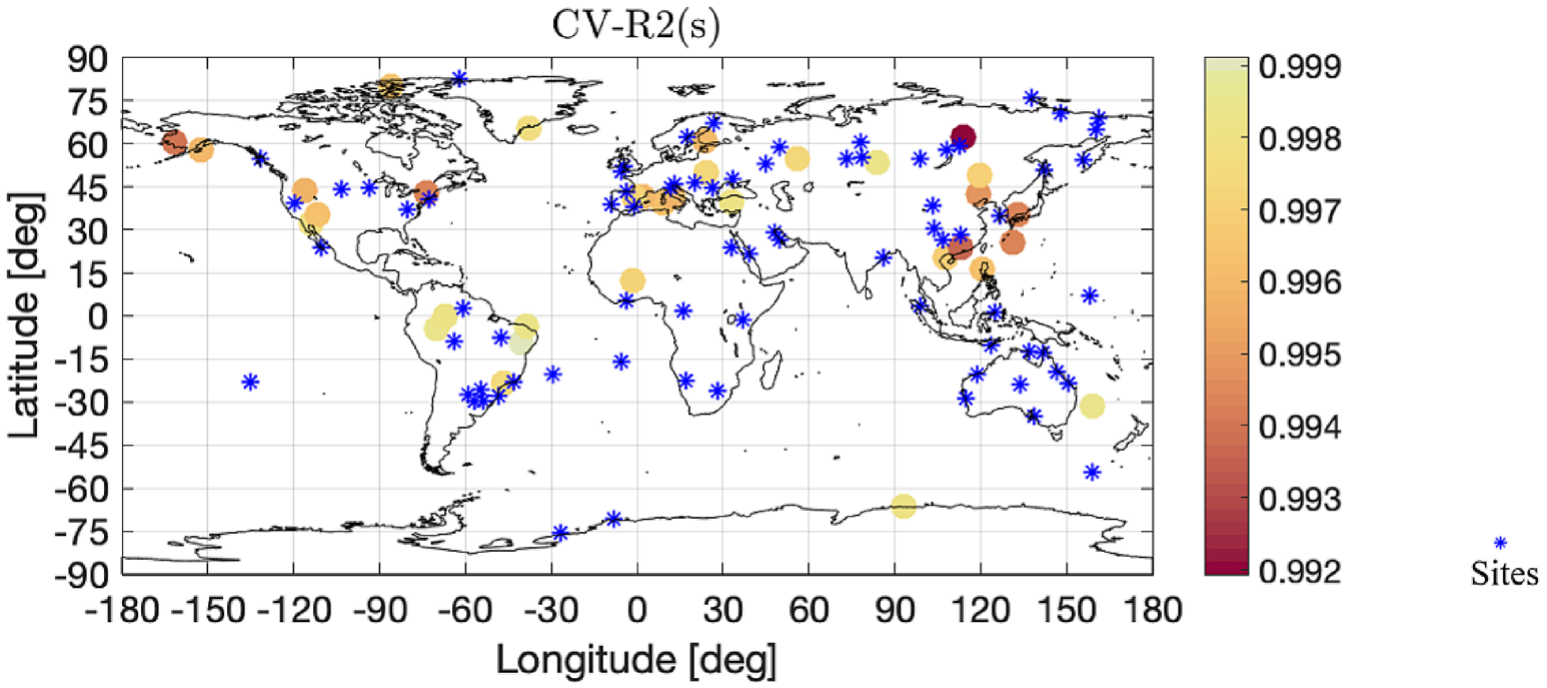
It shows Cross-validation ${\mathrm{R}}^{2}$ with respect to sites $\mathrm{s}$. The larger the value of Cross-validation ${\mathrm{R}}^{2}$, the lighter the color on the map. Sites used for model estimation are marked with blue stars. The values of Cross-validation ${\mathrm{R}}^{2}$ are relatively large. The software used to create the maps in the figure legend is Matlab 2018b, and can be downloaded at https://www.mathworks.com/products/new_products/release2018b.html.

In this section, 30% sites are randomly selected as test set, and the remaining 70% sites are used for model estimation as training set. The real value and the predicted value from test set are defined as $y=({y}_{1},{y}_{2},\dots ,{y}_{{n}_{1}})$ and ${y}^{*}=(y{*}_{1},y{*}_{2},\dots ,y{*}_{{n}_{1}})$ respectively, where n1 represents the number of sites in the test set. By comparing the predicted value with the real value for the selected 30% sites, we obtain the cross-validation ${R}^{2}$ of the RAOB data model. Specifically,${R}^{2}=1-\sum_{k=1}^{n1}(y*-y{)}^{2}/\sum_{k=1}^{{n}_{1}}{(y-\sum_{k=1}^{{n}_{1}}{y}_{k}/{n}_{1})}^{2}$. We show the fitting criteria ${R}^{2}$ with respect to barometric pressure $h$, time $t$, and launching site ${\varvec{s}}$, respectively, in Figs. 4 and 5. Note that, due to the continuous barometric pressure levels, we divide the pressure domain $[\mathrm{50,925}] \; \text{hPa}$ into 20 equally intervals, and calculate the cross-validation ${R}^{2}$ within these intervals. Therefore, the color bar, of the left part of Fig. 4, represents the number of data in each interval.

The left part of Fig. 4 shows that as the altitude increases, the cross-validation ${\mathrm{R}}^{2}$ becomes smaller. In general, excluding the four upper-altitude intervals, the criteria ${\mathrm{R}}^{2}$ higher than 0.96 is achieved, indicating the high accuracy of model prediction. The right part of Fig. 4 shows that ${\mathrm{R}}^{2}$ fluctuates randomly over time. According to Fig. 5, we find that the accuracy of the prediction is higher in the densely site-located areas; otherwise the accuracy of the prediction is lower.

### Observation gap analysis

A typical use of the estimated model is to gather information on atmosphere where no measurement are available. The average $\mathrm{O}-\mathrm{B}$ profile and the average standard deviation profile are given by

7 $$\begin{array}{c}\widehat{\mathrm{e}}\left(\mathrm{h}\right)=\Phi \left(\mathrm{h}\right)\overline{\mathrm{z} },\end{array}$$

8 $$\begin{array}{c}{\upsigma }_{\widehat{\mathrm{e}}}\left(\mathrm{h}\right)=\Phi \left(\mathrm{h}\right)\overline{\mathrm{Var }\left(\mathrm{z}\right)}\Phi {\left(\mathrm{h}\right)}^{{{\prime}}},\end{array}$$

where $\overline{\mathrm{z} }$ is the average of latent space–time variable $\mathrm{z}\left(\mathrm{s},\mathrm{t}\right)$.

**Figure 6:**
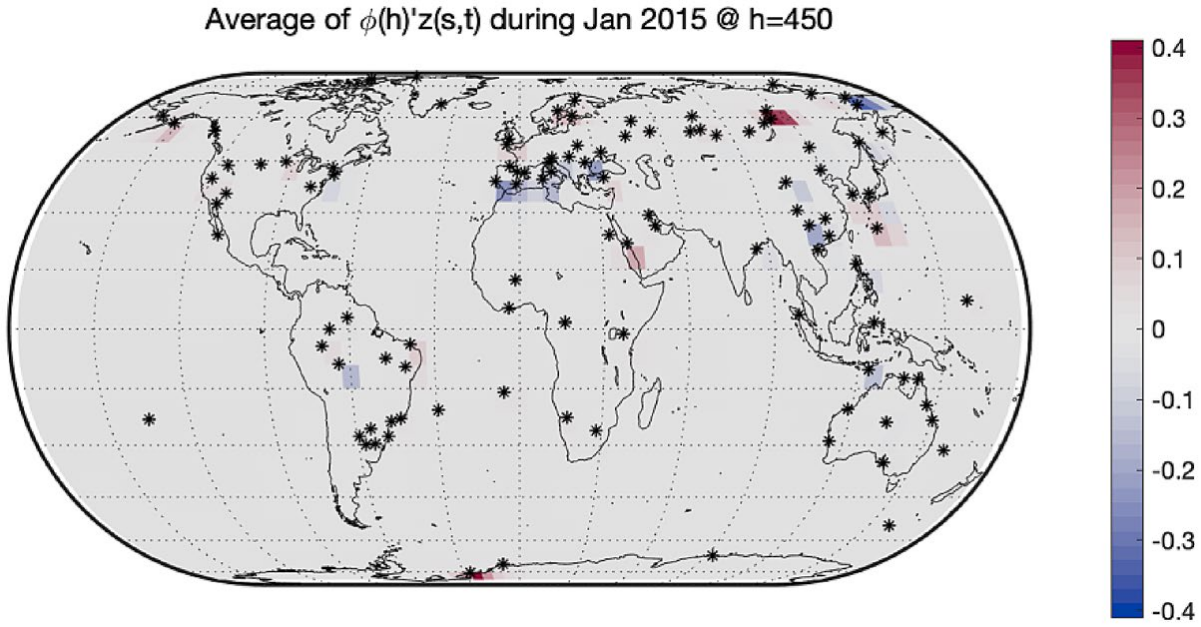
The average difference $\upphi {\left(\mathrm{h}\right)}{{^{\prime}}}\mathrm{z}\left(\mathrm{s},\mathrm{t}\right)$ between RAOB and ERA-Interim in January 2015 at $450 \; \mathrm{ hPa}$. The bigger the gap, the darker the color. All 200 sites are marked with black stars. The differences in areas where there are sampling sites can be estimated well with color marked on the map. The differences in areas far away from sampling sites cannot be estimated well with gray marked on the map. The software used to create the maps in the figure legend is Matlab 2018b, and can be downloaded at https://www.mathworks.com/products/new_products/release2018b.html.

**Figure 7:**
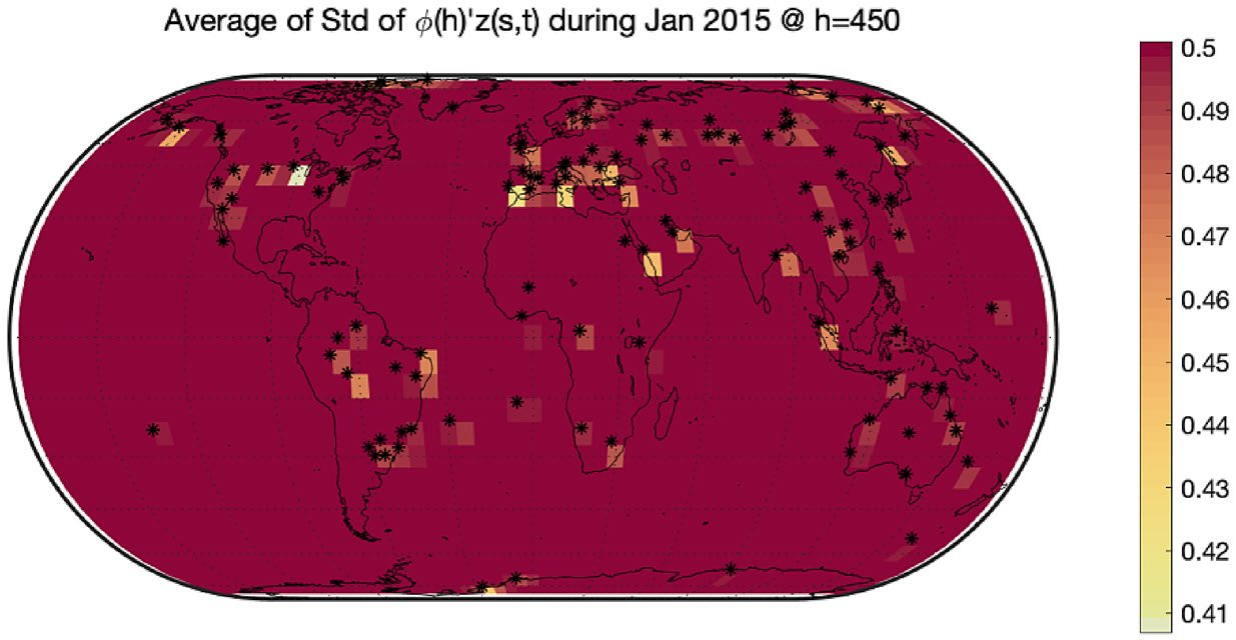
The average standard deviation of difference between RAOB and ERA-Interim in January 2015 at $450 \; \mathrm{ hPa}$. All 200 sites are marked with black stars. The variance of differences in areas where there are sampling sites are very small, while the variance of differences in areas far away from sampling sites are very large. The software used to create the maps in the figure legend is Matlab 2018b, and can be downloaded at https://www.mathworks.com/products/new_products/release2018b.html.

**Figure 8:**
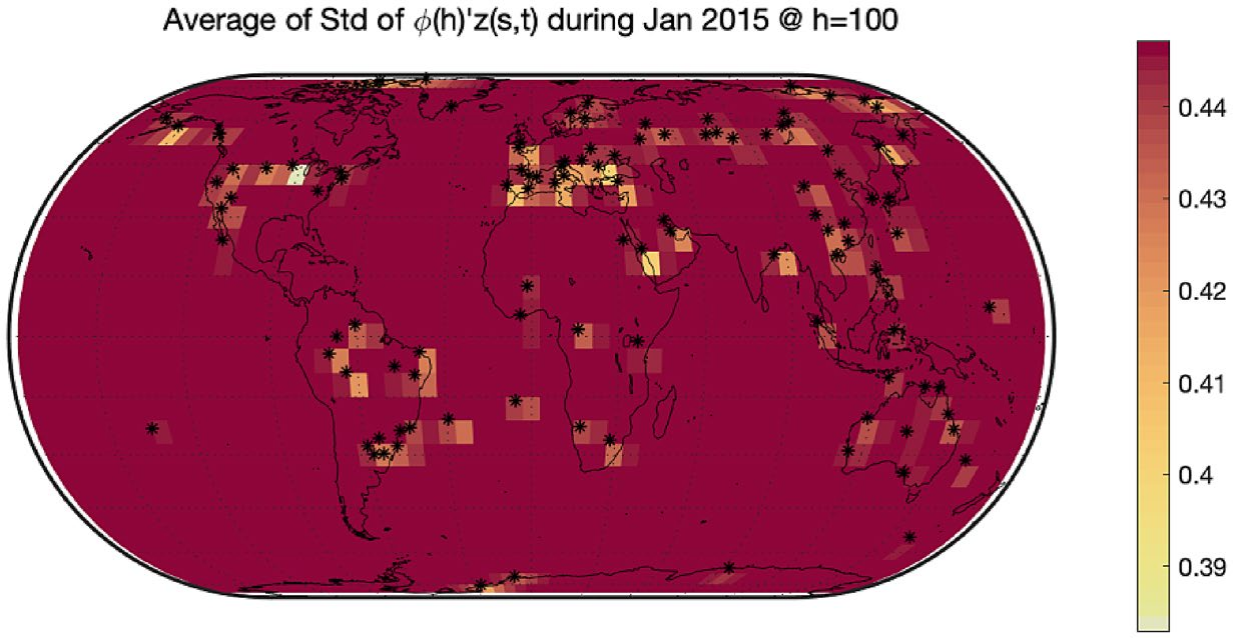
The average standard deviation of difference between RAOB and ERA-Interim in January 2015 at $100 \; \mathrm{ hPa}$. All 200 sites are marked with black stars. The variance of differences in areas where there are sampling sites are very small, while the variance of differences in areas far away from sampling sites are very large. The software used to create the maps in the figure legend is Matlab 2018b, and can be downloaded at https://www.mathworks.com/products/new_products/release2018b.html.

As mentioned in the introduction, the GAIA-CLIM project suggests to consider as observational gaps the areas of the atmosphere where the uncertainty of spatial prediction is higher. Figure 6 shows the average estimates of the difference $\upphi {\left(\mathrm{h}\right)}^{{{\prime}}}\mathrm{z}\left(\mathrm{s},\mathrm{t}\right)$ between the RAOB and ERA-Interim at pressure level $450 \; \mathrm{ hPa}$. As it is expected, the differences in areas where there are sampling sites can be estimated well with color marked on the map. To visualize the changes of the average uncertainty of spatial prediction along different altitudes, we drew Figs. 7 and 8. These two Figures aim to show the value of ${\upsigma }_{\widehat{\mathrm{e}}}\left(\mathrm{h}\right)$ with different h. Figures 7 and 8 depict the average of the standard deviations of the spatial predictions at barometric pressures $450 \; \mathrm{ hPa}$ and $100 \; \mathrm{ hPa}$, respectively. We found that, no matter what the altitude is, the variance of differences in areas where there are sampling sites are very small, while the variance of differences in areas far away from sampling sites are very large. In this way, we explore the underlying mechanism of how the uncertainty changing at various barometric pressure levels.

## Conclusion

Meteorological data such as temperature data of the upper atmosphere is essential for accurate weather forecasting. In this paper, we propose a 4D spatiotemporal model that objectively measures the observation gap of two types of temperature data (RAOB data and ERA-Interim reanalysis data). Based on the $\mathrm{O}-\mathrm{B}$ method, the observation gap is analyzed through the spatial prediction results. The estimated results of the model are of significantly practical interpretation, and the model is meaningful in theory and practice.

From the theoretical point of view, we propose a 4D statistical model on the basis of traditional 3D model. This model not only considers the traditional third dimension of time, longitude and latitude, but also the dimension of altitude. Our model effectively characterizes the vertical profile of the radiosonde data through a linear combination of random components and B-spline curves. We establish the model with these two kind of temperature data from 200 launching sites worldwide in January 2015. The model shows a good model fittingness by the criteria the cross-validation ${\mathrm{R}}^{2}$.

From an application point of view, it can help climate scientists make effective inferences from ERA-Interim reanalysis data to RAOB data. As mentioned before, ERA-Interim reanalysis data is widely available with low-quality; RAOB data with high-quality but can offer a very limited spatial coverage. Through the method we proposed, we can obtain a huge amount of RAOB data with high-quality and can offer a very wide spatial coverage. We explored the underlying mechanism of how the temperature of atmospheric regions changing at different barometric pressure levels, thus providing a meaningful reference for global climate prediction.

## References

[1] Upmanis H, Chen D (1999). Influence of geographical factors and meteorological variables on nocturnal urban-park temperature differences—A case study of summer 1995 in Göteborg, Sweden. Clim. Res..

[2] Lancaster IN (1980). Relationships between altitude and temperature in Malawi. S. Afr. Geogr. J..

[3] Piao S, Cui M, Chen A, Wang X, Ciais P, Liu J, Tang Y (2011). Altitude and temperature dependence of change in the spring vegetation green-up date from 1982 to 2006 in the Qinghai-Xizang Plateau. Agric. For. Meteorol..

[4] Li Y, Ni J (2012). The characteristics of temperature variability with terrain, latitude and longitude in Sichuan-Chongqing Region. J. Geogr. Sci..

[5] Rattigan K, Hill SJ (1987). Relationship between temperature and flowering in almond: Effect of location. Aust. J. Exp. Agric..

[6] Marion GM, Henry GHR, Freckman DW, Johnstone J, Jones G, Jones MH, Levesque E, Molau U, Mølgaard P, Parsons AN, Svoboda J (1997). Open-top designs for manipulating field temperature in high-latitude ecosystems. Glob. Change Biol..

[7] Hammond KA, Szewczak J, Król E (2001). Effects of altitude and temperature on organ phenotypic plasticity along an altitudinal gradient. J. Exp. Biol..

[8] Rojas-Valverde D, Ugalde-Ramírez JA, Sánchez-Ureña B, Gutiérrez-Vargas R (2020). Influence of altitude and environmental temperature on muscle functional and mechanical activation after 30'time trial run. MHSalud.

[9] Li B, Wang Z, Jiang Y, Zhu Z (2018). Temperature control and crack prevention during construction in steep slope dams and stilling basins in high-altitude areas. Adv. Mech. Eng..

[10] Merchant CJ, Paul F, Popp T, Ablain M, Bontemps S (2017). Uncertainty information in climate data records from earth observation. Earth Syst. Sci. Data.

[11] Lizundia-Loiola J, Otón G, Ramo R, Chuvieco E (2020). A spatio-temporal active-fire clustering approach for global burned area mapping at 250 m from MODIS data. Remote Sen. Environ..

[12] Von Clarmann T, Degenstein DA, Livesey NJ, Bender S, Braverman A, Butz A, Compernolle S, Damadeo R, Dueck S, Eriksson P, Funke B (2020). Overview: Estimating and reporting uncertainties in remotely sensed atmospheric composition and temperature. Atmos. Meas. Tech..

[13] Ignaccolo R, Franco-Villoria M, Fassò A (2015). Modelling collocation uncertainty of 3D atmospheric profiles. Stoch. Environ. Res. Risk Assess..

[14] Wang, Y., Finazzi, F., & Fassò, A. D-STEM v2: A Software for Modelling Functional Spatio-Temporal Data. arXiv preprint arXiv:2101.11370 (2021).

[15] Giraldo R, Delicado P, Mateu J (2011). Ordinary kriging for function-valued spatial data. Environ. Ecol. Stat..

[16] Giraldo, R., Delicado Useros, P. F., & Mateu, J. Geostatistics for functional data: An ordinary kriging approach (2007).

[17] Desroziers G, Berre L, Chapnik B, Poli P (2005). Diagnosis of observation, background and analysis-error statistics in observation space. Q. J. R. Meteorol. Soc..

[18] Wang, Y., & Bai, Y. Estimating Observation Error Statistics Using a Robust Filter Method for Data Assimilation. In FSDM, 449–456 (2020).

[19] Dee DP, Uppala SM, Simmons AJ, Berrisford P (2011). The ERA-Interim reanalysis: Configuration and performance of the data assimilation system. Quart. J. R. Meteorol. Soc..

[20] Porcu E, Bevilacqua M, Genton MG (2016). Spatio-temporal covariance and cross-covariance functions of the great circle distance on a sphere. JASA..

[21] Poli, P. *et al.* Pre-assimilation feedback on a Fundamental Climate Data Record of brightness temperatures from Special Sensor Microwave Imagers: A step towards MIPs4Obs? ERA report series (2015).

